# Neutrophils in Intracerebral Hemorrhage: Roles, Mechanisms, and Therapeutic Implications

**DOI:** 10.1155/mi/6650301

**Published:** 2025-12-09

**Authors:** Junzhi Chu, Yingying Qiu, Qiujun Zhou, Jianzhong Yu

**Affiliations:** ^1^ Department of Neurology, Tiantai County Chinese Medicine Hospital, Taizhou, Zhejiang, 317200, China; ^2^ First Affiliated Hospital, College of Medicine, Zhejiang University, Hangzhou, 310003, China, zju.edu.cn; ^3^ Department of Neurology, The First Affiliated Hospital of Zhejiang Chinese Medical University (Zhejiang Provincial Hospital of Chinese Medicine), Hangzhou, Zhejiang, 310006, China, zjhtcm.com

**Keywords:** inflammatory response, intracerebral hemorrhage, neutrophil, neutrophil extracellular traps, prognostic biomarker, therapeutics

## Abstract

Nontraumatic intracerebral hemorrhage (ICH), characterized by bleeding into the brain parenchyma, is a major cause of adult disability and mortality. The pathophysiology of ICH involves complex processes, including mass effect and subsequent inflammatory responses, which cause severe primary and secondary brain damage. As the first responders in neuroinflammatory reactions, neutrophils are rapidly recruited to the hemorrhage site. They interact with other immune cells, release cytotoxic molecules, and significantly exacerbate neuroinflammation. In the acute phase, neutrophils secrete cytokines, chemokines and neutrophil extracellular traps (NETs), which are particularly detrimental to brain tissue. However, in later stages, infiltrated neutrophils can adopt an immunosuppressive phenotype, exerting beneficial effects. Emerging evidence reveals that neutrophils play a multifaceted role in ICH progression, shifting between anti‐inflammatory or pro‐inflammatory phenotypes depending on brain tissue niche. Hence, tuning neutrophils into a beneficial phenotype represents a promising therapeutic strategy for ICH. We conducted a comprehensive literature search in PubMed and Web of Science databases for relevant studies published up to July 2025, using keywords including “intracerebral hemorrhage (ICH),” “neutrophil,” “inflammation,” “neuroinflammation,” “ neutrophil extracellular traps (NETs),” “treatment,” “therapy,“ and “therapeutics.” In this article, we explore the roles of neutrophils in ICH, encompassing their recruitment, activation mechanisms, interactions with other immune cells, and impact on neuroinflammation and neuronal injury. Furthermore, we discuss therapeutic strategies targeting neutrophil‐mediated pathways in ICH, highlighting potential avenues for future research and clinical intervention.

## 1. Introduction

Intracerebral hemorrhage (ICH) is a severe cerebrovascular disorder characterized by bleeding into the brain parenchyma, often resulting from the rupture of small cerebral arteries. The annual incidence of ICH ranges from 12 to 15 cases per 100,000 individuals [1]. In Western countries, it accounts for ~10%–15% of all strokes, while in China it is even higher (18.8%–47.6%). ICH is associated with disproportionately high rates of morbidity and mortality compared to ischemic stroke, with estimates suggesting up to 40%–50% of patients succumb to the condition within the first 30 days, and many survivors facing long‐term disabilities [2]. The etiology of ICH is multifactorial, with hypertension being the most common risk factor. Other contributing factors include cerebral amyloid angiopathy, anticoagulant use, and vascular malformations. Hypertension‐related hemorrhages frequently occur in deep subcortical brain regions, such as the basal ganglia, cerebellum, and pons, while nonhypertensive mechanisms of ICH predominate in the lobar location [3]. Notably, acute lobar cerebral hemorrhages present a distinct clinicopathological feature and a more severe early prognosis compared to deep subcortical hemorrhages, including a higher rate of in‐hospital mortality [4–6]. Mounting evidence has suggested that cerebral amyloid angiopathy is one of the major causes of acute lobar cerebral hemorrhages [5, 7].

Prognosis prediction of ICH is crucial for clinical management and patient outcomes [8, 9]. For instance, intraventricular extension of ICH has been well‐established as a determinant of poor prognosis [10]. As demonstrated in earlier studies, intraventricular hemorrhage is strongly associated with increased mortality and worse functional outcomes [11, 12]. It is also identified as a powerful independent predictor of in‐hospital mortality (OR = 24.74) in patients with thalamic hemorrhage [11]. Although current studies did not systematically explore ventricular extension across all hemorrhage subtypes, its significant effect on early outcomes reinforces the importance of monitoring and addressing this complication in clinical practice [12]. So far, the Hemphill‐ICH score—incorporating GCS, age, hematoma volume, intraventricular extension, and location—is a well‐validated tool with high predictive accuracy (AUC = 0.80) [13]. Both the ICH score and GCS are objective measures strongly associated with functional outcomes and mortality [13–15].

Treatment of ICH poses significant challenges due to the rapid onset and subsequent catastrophic neurological damage [16]. Surgical interventions, such as hematoma evacuation or decompressive craniectomy, are often considered to alleviate intracranial pressure [17]. Research into pharmacological treatments for ICH is ongoing, with efforts focused on mitigating secondary brain injury mechanisms, such as inflammation, oxidative stress, and neuronal apoptosis [18]. Current understanding about the pathophysiological and molecular mechanisms of ICH remains limited, and effective treatments are urgently needed to improve patient outcomes.

Neutrophils are major participants in nonspecific immune responses, acting as “first responders” to brain injury [19]. Like other types of leukocytes, neutrophil originates from hematopoietic stem cells located in the bone marrow [20]. Following a series of differentiation steps (granulopoiesis), neutrophils are released into the bloodstream as mature cells with a half‐life of 6–8 h. Mature neutrophils circulate in the bloodstream, constantly surveying for signs of infection. When they encounter a pathogen or inflammatory signal, they adhere to the endothelium of blood vessels and migrate toward the targeted site through a process called chemotaxis, which is guided by chemical gradients of molecules released by injured tissues or immune cells [21]. Upon reaching the infection site, neutrophils use their phagocytic machinery to engulf pathogens. This process involves the formation of pseudopods that extend around the microbe, eventually enclosing it within a phagosome. Inside the phagosome, the neutrophil employs multiple antimicrobial mechanisms, such as fusion with lysosomes containing digestive enzymes and production of reactive oxygen species (ROS) [22, 23]. Neutrophils are indispensable components of the innate immune system, adept at recognizing, engulfing, and destroying pathogens through a variety of mechanisms. Their ability to mount rapid responses and coordinate with other immune cells makes them critical in maintaining homeostasis and defending against infections in the human body.

**Figure 1:**
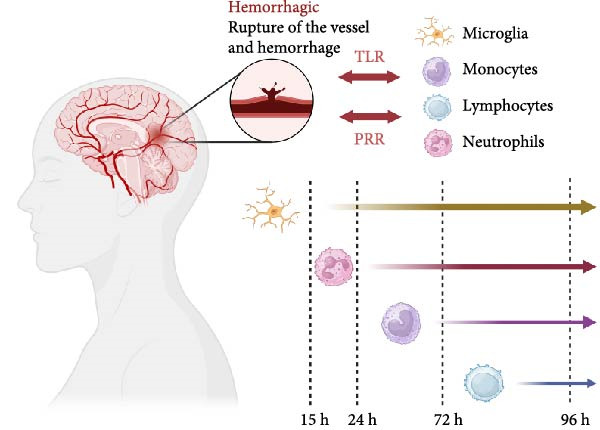
Immune cell activation timeline after intracerebral hemorrhage. Intracerebral hemorrhage induces the activation of microglia, allowing various immune cells to migrate from the microvasculature into the brain parenchyma, with neutrophils being the earliest to undergo migratory activation.

Although most studies have focused on the prognostic value of neutrophils‐to‐leukocytes ratio in ICH, mounting experiments also demonstrate the crucial role of leukocyte‐centered inflammation in ICH‐triggered secondary brain damage. Typically, neutrophils expand within a few hours, damage the blood–brain barrier (BBB), migrate into the brain hemorrhage site from peripheral blood, and release proteases (such as matrix metalloproteinase (MMP)‐3 and 9), cytokines (such as IFN‐γ and tumor necrosis factor (TNF)‐α), and detrimental substances (such as ROS), as well as neutrophil extracellular traps (NETs) (Figure 1) [23–25]. Despite the adverse effects of neutrophils in the acute phase of ICH, their immunosuppressive functions in the later stages may help contain the inflammation and improve patient outcomes [26, 27]. Hence, a comprehensive and detailed understanding of the neutrophil‐associated pathophysiological process in ICH may facilitate the development of novel therapeutics. In this review, we provide a comprehensive overview of the current knowledge on the dual roles of neutrophils in ICH. To ensure a systematic approach, we performed a literature search across PubMed and Web of Science electronic databases for original articles and reviews published until July 2025. Search terms included combinations of “intracerebral hemorrhage,” “ICH,” “neutrophil,” “neuroinflammation,” “neutrophil extracellular traps,” “NETs,” “treatment,” “therapeutics,” and “therapy.” Relevant studies were selected based on their focus on mechanistic insights, therapeutic potential, and relevance to the ICH pathology. This review synthesizes the evidence from these selected studies to elucidate the mechanisms of neutrophil‐mediated injury and protection and to discuss emerging neutrophil‐targeting therapeutic strategies.

## 2. Review Methodology

### 2.1. Literature Search Strategy

A systematic literature search was conducted to identify all relevant publications pertaining to neutrophils in ICH. The electronic databases PubMed and Web of Science were queried for articles published from inception until July 2025. The search strategy employed a combination of Medical Subject Headings (MeSH) terms and free‐text keywords, including: (“intracerebral hemorrhage” OR “ICH” AND (“neutrophil”) AND (“inflammation” OR “neuroinflammation” OR “NETs” OR “neutrophil extracellular traps” OR “therapy” OR “treatment” OR “therapeutics”).

### 2.2. Study Selection and Eligibility

The retrieved articles were screened by title and abstract to assess their relevance to the review’s scope. Full‐text articles were then reviewed for inclusion. Eligible study types included original research articles (in vivo and in vitro), review articles, meta‐analyses, and clinical trials. Articles were included if they focused on the mechanisms of neutrophil recruitment, activation, or function in ICH or discussed therapeutic interventions targeting neutrophils in ICH models or patients. Non‐English articles and studies not directly related to ICH or central nervous system (CNS) pathology were excluded.

### 2.3. Data Synthesis

Due to the narrative nature of this review, a qualitative synthesis approach was adopted. The selected literature was analyzed to summarize current understanding, identify key mechanisms, and discuss controversial findings and future directions in the field of neutrophil biology in ICH.

## 3. Neutrophil Recruitment and Activation in Brain

Under normal conditions, the CNS has established an immunosuppressive brain microenvironment, known as “immune privilege,” which involves decreased expression of major histocompatibility (MHC) antigens, the secretion of immunosuppressive molecules including TGF‐β and interleukin‐10 (IL‐10), and induction of apoptosis of infiltrated inflammatory cells by increased expression of Fas ligand [28, 29]. Since antigen‐presenting dendritic cells (DCs) are absent, immune responses only occur in the brain after peripheral antigen presentation [30, 31]. One study showed that when the mouse hippocampus was injected with pro‐inflammatory cytokines, neutrophils tended to circulate in microvessels instead of extravasating into the surrounding brain tissue [32–34]. The reluctance of neutrophil infiltration in the CNS was described as “intrinsic resistance.” Nevertheless, over time, inflammatory responses diminish these intrinsic defenses, eventually making the CNS “permissive” to the entry of immune cells such as neutrophils [35]. Central to neutrophil activation are pattern recognition receptors (PRRs), especially the toll‐like receptors (TLRs), through which conserved microbial motifs are recognized [36, 37]. For instance, TLR2 detects bacterial lipoproteins, activating neutrophils via NF‐κB and MAPK pathways, essential for neutrophil survival and cytokine production [38, 39]. Priming, facilitated by agents like TNF, enhances neutrophil responsiveness to subsequent stimuli, augmenting their antimicrobial functions while potentially exacerbating tissue damage in chronic inflammation [40].

**Figure 2:**
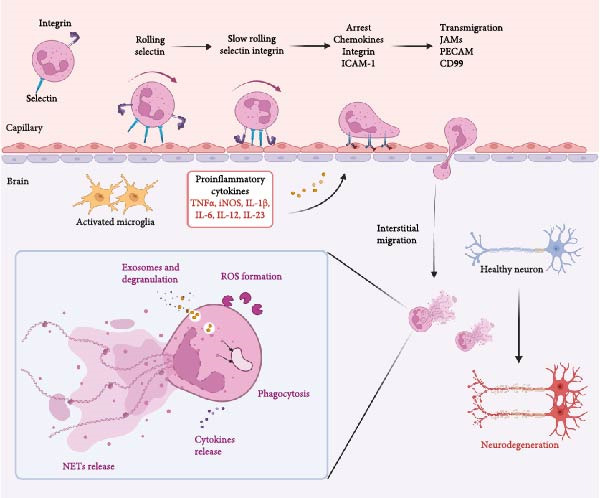
The recruitment of neutrophils involving multiple adhesion steps: upon attachment with endothelium, the surface selectins of neutrophils interact with corresponding ligands to facilitate their rolling. Rolling neutrophils release proinflammatory substances promote the formation of an inflamed environment. Then, integrin interaction with ligands mediates the slow rolling and arrest of neutrophils. Arrested neutrophils release chemokines and highly express ligands, such as icam‐1. Next, the neutrophils transmigrate from endothelium cell–cell junctions into the blood–brain barrier, causing neuronal damage. The killing mechanisms of neutrophils include phagocytosis, generation of ROS, degranulation, and release of nets.

The recruitment of neutrophils to the CNS following ICH is an intricate process orchestrated by various chemotactic signals and adhesion molecules. Upon hemorrhage, blood components like hemoglobin and their degradation products, including iron and heme, act as potent chemoattractants, promoting neutrophils to migrate across the BBB into hemorrhagic parenchymal sites [41, 42]. Inflammatory signals such as IL‐1β, TNF, and granulocyte colony‐stimulating factor (G‐CSF) not only regulate neutrophil activation but also extend their lifespan by inhibiting apoptosis [43]. This prolongation of neutrophil survival is crucial for sustained antimicrobial activity and effective resolution of infection. Previous studies have revealed that cytokines like TNF and IL‐1β significantly increase neutrophil survival rates, ensuring prolonged defense against pathogens and facilitating the orchestration of immune responses [44, 45]. As observed in autopsy samples from human subjects, neutrophils migrate to injury sites ~15 h after the onset of stroke [46, 47]. They initially adhere to the walls of microvascular and microlymphatic vessels, infiltrate into hemorrhagic brain parenchyma at 1–2 days poststroke. After 2–3 days, mononuclear phagocytes begin to enter [48]. Once within the brain tissue, neutrophils become activated by interacting with local endothelial cells (ECs), astrocytes, and microglia. Activation triggers the release of ROS and pro‐inflammatory cytokines (such as TNF‐α and IL‐1β), contributing to neuronal damage and oxidative stress (Figure 2).

## 4. Phagocytosis, ROS Production, and Degranulation of Neutrophils in ICH

Neutrophils play vital roles in innate immune responses during ICH, including phagocytosis, microbial killing, and degradation [49]. They employ opsonic receptors like FcγRs and C‐type lectin receptors to recognize as well as engulf pathogens or cellular debris [50]. This process leads to the formation of phagosomes, where the engulfed material is contained [51]. Neutrophil phagocytosis is exceptionally fast, typically taking less than 20 s to uptake IgG‐opsonized particles [52]. After engulfment, the phagosome matures by fusing with preformed granules within the neutrophil [53]. These granules are rich in hydrolytic enzymes and components of the NADPH oxidase (NOX) complexes, which are crucial for pathogen killing. Unlike macrophages, neutrophils maintain a neutral pH within their phagosomes, which is influenced by their robust oxidative burst [54]. However, in situations like thrombo‐hemorrhagic vasculitis or ICH, where there is extensive tissue damage and inflammation, the process of detrimental phagocytosis may occur [55]. This happens when neutrophils encounter large surfaces coated with immune complexes or complement deposits, which they cannot completely engulf. This incomplete engulfment triggers the secretion of oxidative products and granule contents outside the neutrophil, contributing to further tissue injury and inflammation [56]. Overall, while neutrophil phagocytosis is crucial for defense against pathogens and debris, its dysregulation in conditions like ICH can exacerbate tissue damage through the release of cytotoxic substances and oxidative stress. Understanding these processes is crucial for identifying targeted therapies aimed at mitigating inflammation and improve outcomes for ICH patients.

Another primary mechanism by which neutrophils contribute to neuronal injury in ICH is the production of ROS [21]. ROS production in neutrophils begins with the assembly of functional NOX at the cell surface, where cytoplasmic components (such as p40phox, p67phox, and p47phox) translocate to join membrane‐bound components (such as p22phox and gp91phox), in conjunction with Rac2 or Rac1 [41, 57]. This assembly is crucial for the generation of superoxide anions, initiating the respiratory burst and subsequent rapid ROS production [58]. Myeloperoxidase (MPO), found in primary granules of neutrophils, further promotes the production of hypochlorous acid (HOCl) by catalyzing chloride ions and hydrogen peroxide, which can overwhelm endogenous antioxidant defenses and induce DNA damage, protein oxidation, and lipid peroxidation in surrounding glial cells and neurons [59]. While ROS are essential for pathogen killing, their overproduction may result in oxidative stress and tissue damage. In conditions like ICH, where there is significant tissue injury and inflammation, neutrophil‐derived ROS may aggravate damage to surrounding tissues [27, 41]. Understanding the regulation of ROS production in neutrophils is essential for devising strategies to modulate their activity and reduce inflammatory responses.

In addition, activated neutrophils can exacerbate brain damage through degranulation. They can release four types of granules, which include specific granules containing lysozyme, collagenase, and lactoferrin (LTF), azurophilic granules that contain bactericidal factors (such as heparin‐binding protein, defensins, cathepsins, elastase, and MPO), secretory vesicles, and gelatinase granules [53, 60]. These granules further release proteolytic enzymes to facilitate neutrophil mobilization and to enhance neutrophil adhesion to endothelium. For instance, MMP‐9 contained in tertiary granules can participate in angiogenesis, vascular remodeling, and the digestion of basement membranes [61], thereby compromising the integrity of neurons. Neutrophils are also capable of synthesizing and releasing various cytokines and chemokines, which are critical in regulating inflammatory processes [62]. Among these, CXC‐chemokine ligand 8 (CXCL8, also known as IL‐8) stands out as a potent chemoattractant that recruits neutrophils to sites of infection. Its release from neutrophils is crucial for initiating the inflammatory cascade by attracting other immune cells to the site of injury or infection [63]. Likewise, CCL20 can promote the recruitment and activation of immune cells, aiding in the establishment of an effective immune response [64]. Besides, cytokines stored in secretory granules, such as interleukin 1‐receptor antagonist (IL‐1RA) and tumor necrosis factor‐related apoptosis‐inducing ligand (TRAIL), may play opposite roles in neutrophil‐mediated inflammation. IL‐1RA acts as a negative regulator of IL‐1 signaling, dampening excessive inflammation and preventing tissue damage [62]. TRAIL, on the other hand, induces apoptosis in infected or damaged cells, helping to limit the spread of pathogens and promoting tissue repair [65]. Furthermore, neutrophils also facilitate the resolution of inflammation by producing lipid mediators, including lipoxins, protectin D1, and resolvins (resolvin D1, E2, D2, and E1) [66]. These lipid mediators have anti‐inflammatory properties and are of great significance for resolving inflammation by facilitating the clearance of inflammatory debris and stimulating tissue repair [66]. Lipoxins, for example, inhibit neutrophil infiltration and promote the removal of apoptotic neutrophils (ANs) by macrophages, thus preventing prolonged tissue damage [67]. Resolvins and protectins D1 further contribute to resolution by limiting neutrophil activation and promoting the restoration of tissue homeostasis.

## 5. NETs in ICH

NETs have emerged as a significant topic in neutrophil biology, with a particular focus on their role in various pathological conditions, including ICH. Initially identified by Zychlinsky and his colleagues in 2004, NETs consist of a mixture of chromatin fibers studded with antimicrobial enzymes and peptides including MPO and neutrophil elastase (NE) [68, 69]. These structures are formed through a process termed NETosis, a unique form of cell death distinct from apoptosis, characterized by the release of granule contents and decondensed chromatin into the extracellular space [70]. NETosis can be broadly classified into suicidal and vital NETosis [71]. Suicidal NETosis is triggered by stimuli such as IgG–Fc receptors, TLRs, and cytokines, involving a complex signaling cascade leading to elevated ROS production [71]. This form of NETosis is mediated through Raf/MEK/ERK pathway, resulting in calcium transfer from the endoplasmic reticulum to the cytoplasm, activation of the NOX complex, and subsequent ROS production. ROS, along with MPO, induces NE translocation to the nucleus, where NE mediates histone citrullination, chromatin condensation, and release NETs. Vital NETosis, in contrast, does not depend on ROS and allows neutrophils to retain their integrity and continue functions like phagocytosis and chemotaxis after NET release [70]. Additionally, variations of NETosis include ROS‐dependent mitochondrial NETosis, which utilizes mitochondrial DNA to form NETs, and Gasdermin D (GSDMD)‐mediated NETosis [72]. The diverse stimuli and mechanisms underlying NET formation underscore the complexity of this immune response.

During ICH, NETs exert dual effects, contributing both to host defense and to pathological damage [73, 74]. On the one hand, NETs markedly enhance inflammation post‐ICH. Components of NETs, such as histones, DNA, and citrullinated proteins, exert pro‐inflammatory effects. They activate TLR4 on neutrophils, inducing a cascade of inflammatory responses, including exocytosis, ROS production, and further NET formation [75, 76]. NETs also stimulate the secretion of pro‐inflammatory chemokines like IL‐8 and the B‐cell activating factor (BAFF), amplifying the inflammatory milieu [77, 78]. Oxidative stress is a critical factor in NET formation and its subsequent effects on ICH pathology. ROS production, necessary for NETosis, is elevated in the inflammatory environment post‐ICH. This ROS surge contributes to EC damage, disrupts the BBB, and promotes edema around the hematoma [79]. NETs exacerbate these processes by interacting with ECs, leading to increased permeability and potential rebleeding. NETs also influence the resolution of hematoma and the integrity of the BBB in ICH [80]. They contribute to thrombolytic resistance by binding fibrin, forming DNA‐fibrin tangles that impede the effectiveness of treatments like tissue plasminogen activator (tPA) [81]. This interaction delays hematoma resolution and prolongs inflammation. Furthermore, NETs induce EC necrosis and increase EC permeability, thereby promoting BBB breakdown and edema formation [82].

In the initial stage, NETs promote coagulation and clot retraction, while in subsequent stages, they exacerbate vascular edema through sustained inflammatory responses and BBB disruption [74, 82]. Typically, perilesional edema (PHE) progresses through stages influenced by NETs [82, 83]. NET‐induced MMP activity further degrades tight junction proteins, undermining BBB integrity and contributing to prolonged edema and secondary brain injury [84]. Physiologically, NETs serve to trap and kill pathogens, forming physical barriers that prevent microbial spread. However, overproduction of NETs can lead to autoimmunity and tissue damage. In ICH, excessive NETs amplify local inflammation, induce thrombosis, and cause endothelial damage, compounding the injury caused by the initial hemorrhage [85]. Effective clearance of NETs is crucial to mitigate their harmful effects [86]. DNases, particularly DNase I and DNase II, play a central role in degrading extracellular DNA [87, 88]. Macrophages also facilitate the clearance of NETs through phagocytosis and the secretion of DNases, helping to restore tissue homeostasis [89]. NETs represent a double‐edged sword in the context of ICH. While they constitute a key component of immune defense, their excessive formation and persistence can aggravate brain injury through inflammatory and oxidative mechanisms, thrombolytic resistance, and BBB disruption. Understanding the precise dynamics of NETs formation and clearance in ICH is essential for developing targeted therapeutics to alleviate their destructive effects while preserving their protective functions.

## 6. Interaction Between Neutrophils and Other Cells in ICH

Primary injury in ICH arises from the physical disruption and mass effect of brain tissues, followed by secondary damage driven by neuroinflammatory responses and the release of clotting agents [19]. Resident immune cells like astrocytes and microglia, along with circulating inflammatory cells such as lymphocytes, macrophages, and neutrophils, play a central role in the pathogenesis of ICH [90]. The formation of hematoma in ICH triggers microglial activation, promotes the release of chemoattractants, leading to EC activation and BBB disruption [91]. This allows neurotoxic substances and immune cells (adaptive and innate immune cells) to infiltrate the brain parenchyma, exacerbating secondary brain damage [92]. The intricate crosstalk between immune cells in ICH underscores the complexity of the inflammatory responses. Interactions between microglia, astrocytes, macrophages, lymphocytes, and neutrophils are crucial for both promoting and resolving inflammation, which has drawn increasing attentions recently.

In ICH, microglia respond rapidly within minutes of injury [93]. While monocytes are recruited from peripheral blood, through the interaction of their surface receptors like CCR2 and CXCR2 with chemotactic factors including CCL2 and CXCL12 released from the damaged tissue [92]. They infiltrate the hemorrhagic site after 24–48 h, usually differentiating into macrophages. These immune cells can be polarized into pro‐inflammatory M1 or anti‐inflammatory M2 phenotypes. Specifically, M1 macrophages/microglia, induced by lipopolysaccharide (LPS) and IFN‐γ, generate chemokines (like CC‐2), inflammatory cytokines (like IL‐12, IL‐1β, IL‐6 and TNF‐α), as well as mediator agents (like nitric oxide [NO]), leading to early tissue damage [94]. In contrast, M2 macrophages/microglia, induced by IL‐13 and IL‐4, often release anti‐inflammatory cytokines (like TGF‐β and IL‐10), colony stimulating factors (CSF), as well as growth factors (like insulin‐like growth factor‐1 (IGF‐1)), to support tissue repair [95, 96]. Lymphocytes, although less defined in their role in ICH, become active 3–4 days post‐injury and are crucial in the adaptive immune response [97]. Th1 lymphocytes facilitate M1 macrophage polarization via pro‐inflammatory cytokines like IFN‐γ, while Th2 cells promote M2 polarization via anti‐inflammatory cytokines such as TGF‐β and IL‐13 [98]. As a consequence, Th17 cells and neutrophils are further recruited by proinflammatory factors secreted by activated M1 macrophages, intensifying the immune response of ICH [99]. During later stages of ICH, M2 macrophages secrete TGF‐β and IL‐10 to alleviate inflammatory responses and facilitate wound healing. Astrocytes are usually induced by activated microglia to polarize into an A1 neurotoxic phenotype by releasing C1q, TNF‐α, and IL‐1α, promoting neurocytes apoptosis and amplifying inflammatory damage [100, 101]. Reciprocally, they release cytokines (CXCL10, CXCL1, CCL5, CCL2, and IL15) that further activate microglia toward a pro‐inflammatory state. This mutual activation perpetuates the inflammatory cycle and exacerbates injuries.

Neutrophils are among the first responders to ICH. They are recruited to the injury site by signals from activated microglia and damaged tissues. For instance, microglia‐derived leukotriene B4 (LTB4) specifically attracts neutrophils to the hemorrhagic site, exacerbating neuroinflammatory damage [102]. In turn, neutrophils further contribute to the inflammatory environment by releasing proinflammatory cytokines and ROS, thereby aggravating tissue damage. In contrast, microglia‐secreted IL‐27 inhibits neutrophil inflammatory functions, representing a potential counter‐regulatory mechanism [103]. Notably, Resolvin D1, a lipid mediator released by neutrophils, can reprogram microglial immunometabolism [104]. It shifts the metabolic program of microglia from glycolysis to oxidative phosphorylation, supporting their phagocytosis of neutrophils, thereby alleviating brain inflammation, and offering therapeutic potentials by modulating immune balance. Furthermore, neutrophils can modulate the activation and function of lymphocytes, altering adaptive immune responses and contributing to the chronic inflammatory state observed in the late stages of ICH recovery [105]. This highlights the importance of balanced immune responses to limit tissue damage while enabling repair.

Pericytes and ECs that constitute the BBB become activated in response to injury signals, upregulating the expression of adhesion molecules like vascular cell adhesion molecule‐1 (VCAM‐1) and intercellular cell adhesion molecule‐1 (ICAM‐1) [106]. VCAM‐1 and ICAM‐1 facilitate the extravasation of circulating immune cells, like neutrophils, into the hemorrhagic site [107]. Under normal conditions, ICAM‐1 and VCAM‐1 are minimally expressed but increase significantly following ICH, promoting immune cell infiltration and inflammation [108]. Understanding these mechanisms may offer novel therapeutic targets for ICH treatment.

## 7. Potential Therapeutics Targeting Neutrophils in ICH

**Figure 3:**
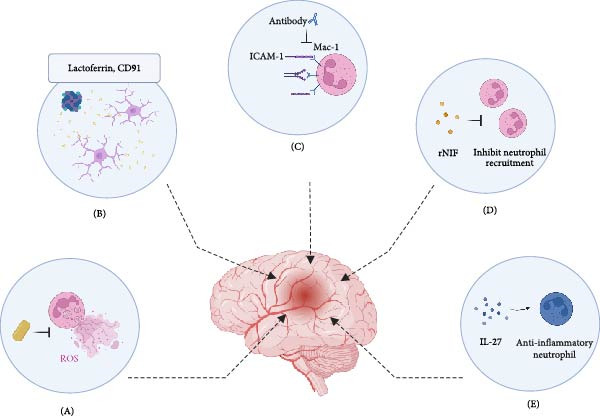
Potential therapeutic strategies targeting neutrophils in ICH. (A) Scavenging ROS and Inhibiting NETosis: edaravone dexborneol reduces ROS and NET formation. (B) Iron chelation and enhancing clearance: Lactoferrin (LTF) binds iron and promotes debris clearance via CD91. (C) Blocking neutrophil adhesion: antibodies against ICAM‐1/Mac‐1 inhibit neutrophil adhesion and transmigration. (D) Directly targeting neutrophils: recombinant neutrophil inhibitory factor (rNIF) inhibits neutrophil trafficking and recruitment. (E) Reprograming Neutrophil Phenotype: IL‐27 promotes anti‐inflammatory neutrophil polarization.

Given the detrimental role of) neutrophils in the pathophysiology of ICH, several interventional strategies have been explored to mitigate neutrophil‐mediated injury and improve outcomes in affected patients (Table 1 and Figure 3).

**Table 1:** Advantages and disadvantages of potential therapeutics targeting neutrophils in ICH.

Therapeutic strategy	Key advantages	Major disadvantages/challenges	References
Edaravone dexborneol (ROS scavenger)	• Reduces brain edema and improves neurological outcomes • Dual inhibitor of NOX and ROS, mitigating oxidative stress and NETosis • Inhibits NLRP3 inflammasome, reducing neuroinflammation	• Narrow therapeutic time window in preclinical models • Efficacy may be limited if administered late in the inflammatory cascade	[109–117]

Lactoferrin (LTF, iron chelation/phagocytosis)	• Promotes hematoma resolution via CD91‐mediated clearance of apoptotic cells • Binds free iron, reducing oxidative stress and ferroptosis • Extended therapeutic window (up to 24 h post‐ICH) in animal studies	• Efficacy dependent on CD91 receptor function • Optimal dosing and delivery methods for clinical use require further investigation	[26, 118–121]

Targeting adhesion molecules (e.g., ICAM‐1)	• Reduces neutrophil infiltration and secondary brain damage in animal models • Antibodies (e.g., anti‐Mac‐1) can work without causing systemic neutropenia	• Clinical translation failure: anti‐ICAM‐1 (enlimomab) worsened outcomes due to immunogenicity • Redundancy in adhesion pathways may limit the efficacy of single‐molecule targeting	[122–135]

UK‐279,276 (rNIF, Mac‐1 inhibitor)	• Well‐tolerated in patients with sustained target saturation • Shows promise in combination with tPA, potentially extending its therapeutic window	• Lack of efficacy as monotherapy: a major clinical trial (ASTIN) was stopped early for futility	[136–139]

Targeting cytokines and NETs	• CXCL1 decoy: innovative strategy to divert neutrophils away from the brain, reducing infarct size • IL‐27 reprograming: skews neutrophils toward a beneficial phenotype, increasing lactoferrin production • DNase I: Degrades NETs and enhances hematoma lysis ex vivo, offering a direct intervention	• IL‐27’s complex role: its pleiotropic nature requires precise modulation to avoid unintended effects • NET inhibition challenges: timing is critical; inhibiting NETs too broadly may compromise host defense, and delivering degrading enzymes (e.g., DNase) to the brain site is difficult	[39, 140–146]

### 7.1. Edaravone Dexborneol

The reduction of ROS is a critical aspect of ICH therapy [109]. Edaravone [110] dexborneol, as a potent ROS scavenger, neutralizes free radicals, reducing oxidative damage and subsequent inflammatory responses. It not only protects neurons but also prevents the exacerbation of brain injury (Figure 3A). It has demonstrated significant therapeutic potential in reducing ICH‐induced brain injury through various mechanisms, including its role as an inhibitor of NOX and ROS. NOX enzymes are major sources of ROS in the brain following ICH. Edaravone dexborneol, alongside other NOX inhibitors such as diphenyleneiodonium and apocynin, effectively inhibits NOX activity, thereby reducing ROS production, mitigating oxidative stress, and preventing further neuronal damage. Notably, ROS production, particularly via NOX, is a key contributor in NETosis, a process by which neutrophils release extracellular traps to aggravate inflammation and disrupt BBB [111]. By inhibiting NOX and ROS, edaravone dexborneol upregulates tight junction‐associated proteins and reduces NETosis, thus mitigating one of the pathways that contribute to secondary brain injury post‐stroke [112]. Oxidative stress plays a critical role in secondary brain injury post‐ICH. Edaravone dexborneol has also been shown to downregulate oxidative DNA damage markers such as apurinic/apyrimidinic abasic sites and 8‐hydroxyl‐2′‐deoxyguanosine [113]. Overall, edaravone dexborneol exhibits potent free‐radical scavenging properties that reduce oxidative injury and subsequent neuronal damage.

In addition, multiple studies have shown that Edaravone dexborneol effectively reduces brain edema and improves neurological outcomes following ICH [114, 115]. For instance, Nakamura et al. [113] demonstrated that systemic administration of Edaravone immediately or 2 h after ICH induction significantly reduced brain effusion and ameliorated neurological damage compared to the control group. It also reduced the volume of brain injury and improved neurologic behavioral deficits in animal models, highlighting its neuroprotective efficacy. A study by Shang [116] and his colleagues utilizing FDG‐PET/CT imaging revealed that edaravone improves perihematomal glucose metabolism, which is crucial for brain recovery post‐ICH. Edaravone‐treated rats exhibited higher FDG uptake in perihematomal regions, indicating improved metabolic activity and reduced brain injury. Edaravone significantly decreases apoptotic cell death, which is associated with improved neurological outcomes and decreased brain edema, further supporting its role in protecting neuronal cells from ICH injury [114]. Edaravone dexborneol also modulates inflammatory pathways. Notably, it inhibits the NLRP3 inflammasome in microglia, resulting in decreased expression of pro‐inflammatory cytokines (IL‐1β, Caspase 1) and NF‐κB, thereby reducing neuroinflammation and neurodegeneration [117]. Overall, Edaravone showed parallel effects when compared with NLRP3 inhibitor MCC950, underscoring its anti‐inflammatory mechanism in ICH treatment. Edaravone dexborneol exhibits significant therapeutic benefits in ICH by reducing brain edema, attenuating oxidative injury, enhancing cerebral metabolism, suppressing inflammatory responses and improving neurological outcomes. Its dual role as an NOX and ROS inhibitor makes it a promising candidate for ICH therapy.

### 7.2. LTF

ICH initiates a cascade of events involving red blood cell (RBC) lysis, leading to cytotoxicity, inflammation, and oxidative stress. Notably, LTF, which is released by neutrophil granules during ICH, is a multifunctional glycoprotein belonging to the transferrin family, primarily known for its iron‐binding properties and its role in innate immunity (Figure 3B) [118]. Under the acidic conditions typical of hemorrhagic brain tissues, LTF can still bind Fe^3+^ with high affinity and efficiently remove free iron in the brain microenvironment with high efficiency [26]. More interestingly, the unique electrochemical properties of LTF protein allow it to easily bind to necroptotic or apoptotic cells or molecules with negative charges [26]. The molecular basis of LTF’s therapeutic effects also involves interactions with scavenger receptor CD91 on microglia/macrophages, crucial for efficient clearance of ANs and hematoma resolution post‐ICH [119]. CD91‐mediated phagocytosis of ANs by microglia/macrophages is impaired in CD91‐deficient models, highlighting the essential role of this receptor–ligand interaction in mitigating ICH‐induced damage. Importantly, LTF’s therapeutic benefits are diminished in CD91‐deficient conditions, emphasizing its dependence on functional CD91 for optimal efficacy in clearing cellular debris and reducing oxidative stress [119].

Recently, increasing efforts have focused on exploring the therapeutic potential of LTF. Zhao [120] and his colleagues utilized an optimized form of LTF, FcLTF, highlighting its efficacy in reducing neurological injury post‐ICH. In animal models, FcLTF attenuates neuronal injury by shielding neurons from RBC‐derived toxins and promoting microglial phagocytosis of apoptotic cells and cellular debris. Moreover, FcLTF’s prolonged therapeutic window of up to 24 h post‐ICH onset suggests greater flexibility in clinical application, potentially extending treatment opportunities beyond current limitations. In addition, a study by Xiao et al. [121] indicated that LTF supplementation offers a promising avenue for therapeutic intervention in diabetic patients experiencing ICH. By inhibiting neuronal ferroptosis, a form of cell death involving iron accumulation, LTF protects neurons from oxidative stress and enhances neurobehavioral recovery in hyperglycemic ICH mouse models. These findings underscore LTF’s role not only in general ICH management but also in addressing specific complications such as those posed by diabetic conditions. Hence, LTF functions as a molecular bridge that couples apoptotic or necroptotic cells and damaged erythrocytes to corresponding receptors on the cell surface of macrophages or microglia and promotes their phagocytic effects. Overall, through the clearance of iron and apoptotic or necroptotic cells, LTF suppresses inflammatory responses and contributes to detoxification and resolution of brain hematoma, providing a novel therapeutic option. Future translational research efforts should focus on refining LTF‐based therapies, exploring combinatorial approaches, and optimizing treatment protocols to enhance efficacy in clinical settings.

### 7.3. Targeting Neutrophil Adhesion Molecules

#### 7.3.1. ICAM‐1

ICAM‐1 has been strongly implicated in the neutrophil‐mediated inflammatory response associated with stroke, both in ischemia and ICH (Figure 3C) [122]. ICAM‐1 is an inducible adhesion molecule expressed on neutrophils, as well as ECs, and other cell types in response to inflammatory stimuli [123]. During stroke, ICAM‐1 expression is upregulated, facilitating the adhesion and transmigration of neutrophils and other immune cells into the brain parenchyma [122], which aggravates inflammation and contributes to the extent of ischemic or hemorrhagic injury. ICAM‐1 has been shown to compromise microcirculation in the brain during ischemic events. A study using ICAM‐1‐knockout mice demonstrated that the absence of ICAM‐1 mitigated ischemic lesion size and preserved microcirculation better compared with wild‐type mice, despite similar levels of granulocyte accumulation [124]. This study indicated that ICAM‐1 plays an important role in microcirculatory failure, contributing to the formation and expansion of ischemic lesions. Intriguingly, ICAM‐1 binds to leukocyte integrins, primarily LFA‐1 (CD11a/CD18), to facilitate the transendothelial migration and adhesion of neutrophils [125]. In ICAM‐1‐deficient mice, transient focal ischemia resulted in significantly reduced infarct volumes when compared with wild‐type mice, indicating that ICAM‐1‐mediated neutrophil adhesion contributes to ischemic damage [126]. In addition, pro‐inflammatory cytokines like TNF‐α and IL‐1β could upregulate ICAM‐1 expression [127]. Lawson et al. knocked out (KO) IL‐1 converting enzyme (ICE) in mice to inhibit the release of mature IL‐1β proteins. They showed that reduced IL‐1β levels in ICE KO mice resulted in decreased ICAM‐1 expression and subsequent leukocyte infiltration in the CNS [127]. Studies in animal models have provided evidence that targeting ICAM‐1 can reduce ischemic injury [126]. For example, administration of ICAM‐1 antibodies or genetic knockout of ICAM‐1 significantly reduced infarct size and improved outcomes in models of focal cerebral ischemia [124, 126]. These studies suggest that interfering with ICAM‐1‐mediated leukocyte adhesion can mitigate the secondary damage caused by inflammation.

Despite promising results in animal studies, clinical trials have faced challenges [128]. The Enlimomab trial, which tested a murine ICAM‐1 antibody in patients with ischemic stroke, showed no improvement in outcomes compared to placebo groups [129]. Patients receiving Enlimomab had higher mortality rates and more adverse events, such as infections and fever [129]. One explanation for the adverse outcomes in the Enlimomab trial is the immunological response triggered by the murine antibody [129, 130]. The human immune system may recognize murine antibodies as foreign antigens, leading to an inflammatory response that exacerbates the injury [130]. This underscores the importance of developing humanized or fully human antibodies for therapeutic use to minimize immunogenicity. ICAM‐1 plays a pivotal role in the inflammatory responses associated with ICH, particularly in facilitating neutrophil adhesion and transmigration, which exacerbates injury. While targeting ICAM‐1 has shown therapeutic potential in animal models, translating these findings to clinical practice has been challenging due to immunogenicity and adverse responses. Future strategies should focus on developing human‐compatible therapies and exploring alternative approaches to modulate leukocyte behavior to harness the benefits while minimizing the risks.

#### 7.3.2. Other Neutrophil Adhesion Molecules

Other strategies targeting neutrophil adhesion molecules have also been explored. For instance, based on the regulatory mechanisms governing neutrophil migration, adhesion and recruitment, L‐selectin, E‐selectin and P‐selectin have also been investigated as potential therapeutic targets. For treatment specificity, monoclonal antibodies, genetic editing, and receptor antagonists or inhibitors are used. Studies have reported that antibodies against E‐selectin and P‐selectin also exhibit therapeutic effects in reducing ischemic injury, BBB leakage, and neutrophil‐mediated inflammatory responses in preclinical models [131]. However, not all adhesion molecules are equally effective targets. Bednar et al. [132] have shown that humanized anti‐L‐selectin monoclonal antibody, DREG200, did not produce significant benefits alone, indicating the need for precise targeting.

### 7.4. Directly Targeting Neutrophils

#### 7.4.1. Mac‐1 (CD11b/CD18)

Mac‐1 (CD11b/CD18) is a key integrin on the neutrophil surface involved in neutrophil adhesion to ECs and migration into tissues during inflammatory responses (Figure 3C) [133]. Targeting Mac‐1 to inhibit neutrophil activity has been identified as a potential therapeutic avenue to alleviate inflammatory damage during ICH. Mac‐1 facilitates neutrophil adhesion to the endothelium as well as transmigration into brain parenchyma by interacting with its ligands, such as ICAM‐1 [134]. Neutrophils are thereby recruited to sites of ischemic or hemorrhagic brain injury, where they contribute to secondary brain damage. In rat models with cerebral ischemia and traumatic brain injury, treatment with antibodies targeting Mac‐1 has been shown to significantly reduce neutrophil accumulation in the brain and mitigate associated inflammation and tissue damage. Clark [135] and his colleagues developed a murine monoclonal antibody to rat Mac‐1 (1‐B6) or anti‐Mac‐1F(ab)2′ fragment, which demonstrated significant reductions in post‐traumatic neutrophil accumulation in the CNS without causing systemic neutropenia. Furthermore, treatment with anti‐Mac‐1 antibodies reduced brain MPO activity, a marker of neutrophil infiltration, by 43% compared to control groups [135]. This reduction in neutrophil accumulation was associated with decreased neuronal damage and improved functional outcomes. These results suggested that targeting Mac‐1 can effectively reduce neuroinflammation and protect brain tissue without compromising the overall immune response.

#### 7.4.2. UK‐279,276

UK‐279,276, also known as recombinant neutrophil inhibitory factor (rNIF), is a glycoprotein derived from hookworms that selectively binds to the CD11b/CD18 integrin on neutrophils (Figure 3D). By targeting Mac‐1, UK‐279,276 has the potential to modulate the neuroinflammatory response in ICH. Mechanistically, UK‐279,276 binds to CD11b/CD18 integrin on neutrophils, inhibiting their ability to adhere to ECs and migrate into the brain tissues, thereby reducing neutrophil accumulation, mitigating the extent of inflammation and subsequent neuronal damage. A multicenter, double‐blind, dose‐escalation study involving 176 patients evaluated the pharmacodynamics, pharmacokinetics, safety, and tolerability of UK‐279,276 in patients with acute stroke [136]. The study found that UK‐279,276 was well tolerated by patients at doses up to 1.5 mg/kg, showing no significant relationship between dose and adverse events. Pharmacokinetics showed nonlinear behavior, and the duration of CD11b saturation was dose‐dependent, achieving over 80% saturation for more than 7 days at the higher doses [136]. This prolonged saturation suggests sustained inhibition of neutrophil activity, potentially reducing neuroinflammation during the critical early period following a stroke. The ASTIN (Acute Stroke Therapy by Inhibition of Neutrophils) study aimed to establish whether UK‐279,276 improves recovery in acute ischemic stroke [137]. Despite the promising preclinical data, the study was terminated early due to lack of efficacy, as UK‐279,276 did not significantly improve recovery compared to placebo [137]. However, a post hoc analysis suggested a modest improvement in patients who were also treated with tPA, indicating a potential synergistic effect when used in combination with thrombolytic therapy [137].

Zhang et al. [138] reported that in a rat model with embolic middle cerebral artery occlusion, UK‐279,276 combined with recombinant human tPA (rhtPA) significantly reduced infarct volume and improved neurological function compared to either treatment alone. The combination therapy also reduced neutrophil accumulation and fibrin deposition in the brain, suggesting the potential for UK‐279,276 to extend the therapeutic window for thrombolytic therapy in acute stroke [138]. Additional animal studies demonstrated that continuous treatment with UK‐279,276 for 48 h following 2 h of MCAO (middle cerebral artery occlusion) significantly reduced infarct volume and neutrophil infiltration in a dose‐dependent manner [139]. The therapeutic time window was found to be within 4 h post‐MCAO, and continuous treatment was necessary for optimal neuroprotection [139]. These findings underscore the importance of early and sustained intervention in mitigating ischemic brain injury. UK‐279,276 shows promise in modulating the inflammatory response in acute stroke by inhibiting neutrophil adhesion and migration. While clinical studies have yielded mixed results, the combination with thrombolytic therapy appears particularly promising, suggesting a potential role for UK‐279,276 in enhancing the efficacy of existing stroke treatments.

In summary, Mac‐1‐targeted therapies represent promising avenues for modulating neutrophil‐driven inflammation in stroke. While UK‐279,276 (rNIF) has shown mixed clinical results, its combination with thrombolytic therapy appears promising. Mac‐1‐targeted therapies have demonstrated significant neuroprotective effects in preclinical studies, highlighting their potential for reducing stroke‐related inflammation and improving outcomes. Further research and clinical trials are needed to fully evaluate the therapeutic potential as well as optimize the use of these interventions in stroke treatment.

### 7.5. Targeting Cytokine, Chemokine, and Their Receptors Associated With Neutrophils

Cytokines and chemokines play crucial roles in the regulation of neutrophil behaviors (Figure 3E). Approaches to manipulate neutrophil activation and expansion by targeting cytokines and chemokines have led to varied outcomes for ICH treatment.

TLR4 is a PRR that plays a pivotal role in the innate immune response by recognizing pathogen‐associated molecular patterns (PAMPs) including LPS [140]. In ICH, TLR4 mediates inflammatory responses that contribute to neuronal damage. TLR4 activation leads to the phosphorylation of mitogen‐activated protein kinases (MAPKs) like ERK‐1/‐2, JNK‐1/‐2, and p38, which then trigger the production of pro‐inflammatory cytokines and inducible NO synthase (iNOS) [39]. These molecules exacerbate neuronal injury by promoting oxidative stress and inflammation. Studies have shown that mice with a loss‐of‐function mutation in TLR4 exhibit protection against ischemic brain damage and retinal ganglion cell (RGC) degeneration following axotomy [141]. The absence of TLR4 results in reduced activation of MAPKs and lower iNOS levels in injured neurons, thereby mitigating neuronal stress responses and enhancing survival. Interestingly, the deficiency in TLR4 also led to an increased presence of MPO + neutrophils and Iba1+ microglial cells, suggesting a compensatory inflammatory response [141]. This points to a complex role of TLR4 in balancing pro‐inflammatory and potentially protective immune activities in the brain. Therapeutically, targeting TLR4 could offer neuroprotection in stroke by reducing detrimental inflammatory responses. However, the challenge lies in modulating TLR4 activity to preserve its protective roles while mitigating its harmful effects. This nuanced approach requires further research to develop selective TLR4 inhibitors or modulators that can provide optimal therapeutic benefits.

CXCL1 is a chemokine that functions as a major chemoattractant for neutrophils [142]. Its elevated levels in the brain and serum following stroke correlate with increased neutrophil infiltration and subsequent inflammation [143]. Diverting neutrophil migration away from the brain using CXCL1 can potentially reduce neuroinflammation and improve stroke outcomes. For instance, Stamatovic [144] and his colleagues reported a novel approach using a CXCL1‐soaked sponge implanted peripherally to attract neutrophils away from the ischemic brain. This strategy significantly reduced brain inflammation, infarct size, and improved neurological deficits and survival in a mouse model of thromboembolic (TE) stroke [144]. The CXCL1 sponge acted as a decoy, diverting neutrophils from the brain to the peripheral site, thereby alleviating the inflammatory burden in the brain. This innovative method underscores the therapeutic potential of manipulating chemokine pathways to modulate immune cell trafficking.

IL‐27 is a cytokine upregulated both centrally and peripherally after ICH [145]. In rodent models, IL‐27 has been shown to modify the maturation of neutrophils in the bone marrow, skewing their function from a pro‐inflammatory/cytotoxic phenotype to a more beneficial one [146]. Mechanistically, IL27 led to a decrease in the production of harmful molecules by neutrophils and an increase in the production of protective molecules such as LTF. The therapeutic potential of IL‐27 in ICH lies in its ability to reprogram neutrophils toward a phenotype that favors neuroprotection and tissue repair. These findings indicated that modulating the IL‐27 pathway to enhance the beneficial functions of neutrophils while suppressing their harmful activities could be a viable therapeutic strategy for ICH.

## 8. Limitations of the Review

While this review provides a comprehensive overview of the multifaceted roles of neutrophils in ICH, it is important to acknowledge its limitations. First, the majority of evidence discussed is derived from preclinical animal studies, which may not fully recapitulate the complexity of human ICH pathophysiology and the subsequent immune response. Translating these findings into clinically effective therapies remains a significant challenge. Second, the review encompasses a broad range of neutrophil‐related mechanisms and therapeutic strategies; consequently, it may not delve into the depth achievable by a review focused on a single specific pathway. Furthermore, like all narrative reviews, the selection and interpretation of literature, despite a systematic search strategy, may be subject to author bias. We did not perform a formal systematic review or meta‐analysis, which limits the ability to quantitatively synthesize evidence or definitively assess the strength of specific associations. Finally, the dynamic and heterogeneous nature of neutrophil phenotypes in ICH is still being unraveled. This review highlights this complexity but also underscores the current lack of standardized definitions and markers for specific neutrophil subpopulations in clinical practice, which hinders the precise targeting of these cells for therapeutic benefit. Future research employing more standardized methodologies and focused on human validation is crucial to bridge these gaps.

## 9. Future Research Directions

Building upon the current understanding outlined in this article, several promising avenues for future research emerge. First, further investigation is needed to elucidate the precise signals and mechanisms that drive neutrophil polarization into pro‐inflammatory (N1) versus immunosuppressive or pro‐repair (N2) phenotypes within the ICH milieu. Identifying specific surface markers and reliable tracking methods for these subpopulations in patients is crucial for developing targeted therapies. Second, the temporal dynamics of NET formation and clearance post‐ICH require deeper exploration to identify optimal therapeutic windows for targeting NETosis without compromising innate immunity. Translational studies focusing on the efficacy and safety of novel NET inhibitors (e.g., DNase I and PAD4 inhibitors) in combination with existing neuroprotective strategies are strongly warranted. Finally, leveraging advanced technologies like single‐cell RNA sequencing and spatial transcriptomics on human ICH samples will be instrumental in mapping the heterogeneity of neutrophil responses and their interactions with other CNS cells, ultimately paving the way for personalized immunomodulatory treatments aimed at improving long‐term functional outcomes after ICH.

## 10. Expert Perspectives and Viewpoints

Beyond the mechanistic insights and therapeutic approaches discussed, our analysis of neutrophil biology in ICH reveals several critical viewpoints that merit emphasis and will likely shape future research directions in this field.

First, we posit that the traditional paradigm of neutrophils as uniformly detrimental responders in ICH requires definitive revision. The emerging evidence of functional heterogeneity and phenotypic plasticity (N1/N2 spectrum) suggests that therapeutic success will depend not on the global suppression of neutrophils, but on the precise reprograming of their activity. The challenge lies in developing strategies that can selectively inhibit their destructive functions (e.g., NETosis and cytotoxic degranulation) while preserving or even enhancing their beneficial roles in debris clearance, inflammation resolution, and tissue repair. This could involve harnessing endogenous modulators like IL‐27 or Resolvin D1 to steer neutrophil differentiation toward a pro‐repair phenotype.

Second, a crucial and often overlooked perspective is the spatiotemporal dimension of neutrophil involvement. The role of neutrophils is not static but evolves dramatically from the hyperacute phase (0–24 h) to the subacute and recovery phases. Therefore, the efficacy of any neutrophil‐targeted intervention is inherently tied to its timing. For instance, blocking initial adhesion might only be effective within a very narrow window post‐ICH, while promoting phagocytic clearance via LTF or modulating phenotypes may be more applicable in the days that follow. Future clinical trials must incorporate rigorous biomarker‐driven patient stratification and time‐window definitions to match the right therapeutic mechanism with the correct pathological stage.

Third, we advocate for a “treat‐and‐repair” combinatorial approach. Simply mitigating the initial neutrophilic insult is likely insufficient for achieving optimal long‐term functional recovery. The most promising future therapies will be those that combine acute neuroprotection (e.g., using edaravone dexborneol to curb oxidative burst) with subsequent pro‐reparative interventions (e.g., using LTF to facilitate hematoma resolution and modulate microglial function). Furthermore, combining neutrophil‐targeted strategies with rehabilitation protocols may synergistically enhance neural plasticity and circuit rewiring.

Finally, from a translational standpoint, we highlight the urgent need for validated, clinically accessible biomarkers of neutrophil activity in ICH patients. While the peripheral neutrophil‐to‐lymphocyte ratio (NLR) is a simple prognostic marker, it fails to capture intracerebral neutrophil heterogeneity or specific pathogenic processes like NETosis. The development of advanced neuroimaging techniques to visualize neutrophil infiltration or NET formation in vivo, or the identification of specific NET biomarkers (e.g., citrullinated histones, MPO‐DNA complexes) in biofluids, would revolutionize patient selection and treatment monitoring, paving the way for truly personalized immunomodulatory therapy in ICH.

## 11. Conclusions

In summary, although the pathophysiology of ICH involves complex immune responses with both harmful and beneficial aspects, the targeting of specific neutrophil subpopulations and NETs offers a novel therapeutic avenue. The current research, though limited, underscores the potential of these strategies to improve outcomes in ICH patients. Further investigation into the roles and mechanisms of neutrophils, particularly the N2 subpopulation and NETs, is essential. By refining our understanding and developing targeted therapies, it is possible to address the challenges posed by ICH, potentially reducing its high mortality rate and improving the prognosis for those affected. The exploration of NET inhibitors in clinical settings is a particularly promising area, with the potential to transform the management of ICH and mitigate its devastating impact. Thus, the future of ICH treatment lies in a nuanced approach that harnesses beneficial immune responses while curbing detrimental effects.

## Ethics Statement

This is a review article and ethical approval is not necessary.

## Consent

The authors have nothing to report.

## Disclosure

All the authors approved the final article.

## Conflicts of Interest

The authors declare no conflicts of interest.

## Author Contributions

Concept and design: Jianzhong Yu. Literature collection and sorting: Junzhi Chu, Qiujun Zhou, and Yingying Qiu. Drafting of the article: Junzhi Chu and Qiujun Zhou. Critical revision of the article for important intellectual content and study supervision: Junzhi Chu.

## Funding

The study was funded by the Zhejiang Provincial Administration of Traditional Chinese Medicine Clinical Research Program (Grant 2023ZL198) and the Zhejiang Provincial Medical and Health Science and Technology Program (Grant 2024KY1835).

## Data Availability

No new data were created for this review.
